# Delayed Postoperative Tension Pneumocephalus Treated With a Subdural Evacuating Port System: A Case Report and Review of the Literature

**DOI:** 10.7759/cureus.32514

**Published:** 2022-12-14

**Authors:** Brandon R Laing, Abhishek Janardan, Ipsit Shah, Abrahim N Razzak, Nathan T Zwagerman

**Affiliations:** 1 Neurosurgery, Medical College of Wisconsin, Milwaukee, USA; 2 Internal Medicine, Medical College of Wisconsin, Milwaukee, USA; 3 Medicine, Medical College of Wisconsin, Milwaukee, USA

**Keywords:** craniotomy, neurosurgery, alternative treatment, neurosurgical emergency, subdural evacuating port system, post-neurosurgery, tension pneumocephalus

## Abstract

Tension pneumocephalus (TP) is a rare neurosurgical emergency due to the rise of intracranial pressure from air in the cranial cavity. Tension pneumocephalus’ clinical presentation ranges from headache, visual alterations, altered mental status, and death. Given its nonspecific clinical presentation, tension pneumocephalus is usually diagnosed via computed tomography (CT) imaging. Open burr hole craniotomy is the preferred treatment method for tension pneumocephalus. Subdural evacuating port system (SEPS) drains have, however, seen increased utilization in neurosurgery due to decreased possibilities for infections, reduced seizure probability, and better outcomes post-surgery, especially for elderly patients.

In this article, we present the case of a 67-year-old female with postoperative tension pneumocephalus after the evacuation of an acute subdural hematoma. The patient became symptomatic from tension pneumocephalus, which was evacuated using a subdural evacuating port system drain. Post-drain placement, the patient had a radiographic and clinical resolution of her tension pneumocephalus.

Thesubdural evacuating port system is a useful adjunctive tool for treating tension pneumocephalus.Given the favorable characteristic profile of subdural evacuating port system drains compared to open surgical modalities, further inquiry should be pursued to analyze the feasibility of establishing subdural evacuating port systems as a less invasive treatment alternative.

## Introduction

Pneumocephalus is defined as the presence of air in the intracranial space within the brain parenchyma, dural spaces, or ventricular cavities [1]. This is commonly seen after head/facial trauma or neurosurgical interventions. Pneumocephalus can be classified as acute (less than 72 hours) or delayed (more than 72 hours) [1,2]. Tension pneumocephalus (TP), specifically, is a rare case when there is continued accumulation of intracranial air caused by a “ball valve” mechanism leading to a mass effect on the brain [2]. While pneumocephalus may be asymptomatic, TP is considered a neurosurgical emergency with symptoms of headache, seizures, and altered mental status [2-4]. Without treatment of the rapid expansion of the trapped air cavity within a cranial vault, permanent neurological deterioration or death can occur [3,4]. There has been no widespread study on its incidence given the rarity of TP; however, one review denotes 11 cases being reported [5]. Due to this, knowledge of the risk factors and radiographic and clinical signs associated with TP becomes crucial for early identification and treatment. Typical management involves a combination of surgical evacuation of intracranial air, Trendelenburg positioning, administration of oxygen, repair of bone/dural defects, and placement of a drain [6]. In this article, we present a case of postoperative tension pneumocephalus successfully managed using a subdural evacuating port system (SEPS) drain.

## Case presentation

**Figure 1:**
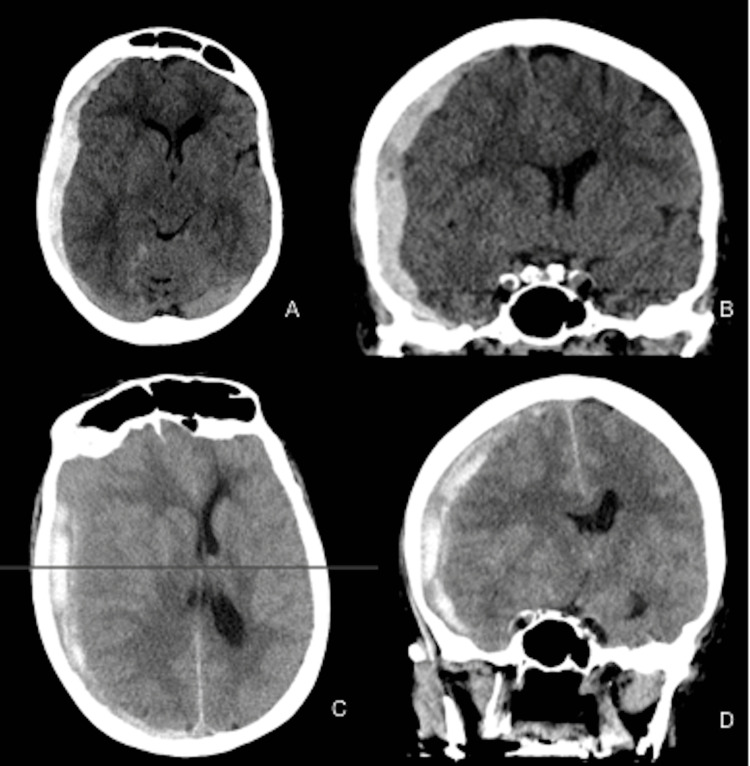
Computed tomography imaging of the head The top row shows axial (A) and coronal (B) views on the initial presentation. The bottom row shows axial (C) and coronal (D) views on re-presentation with acute neurological decline.

The patient is a 67-year-old female with a past medical history of hypertension and rheumatoid arthritis who presented with an acute onset of headache, nausea, and vomiting. On presentation, the patient had a Glasgow Coma Scale (GCS) score of 15 without any neurological deficits. Computed tomography (CT) of the head and CT angiogram (CTA) of the head and neck were ordered on initial evaluation. CT scan of the head displayed a 9-mm acute right subdural hematoma with 7 mm of midline shift (Figure 1). Due to the spontaneous hemorrhage and lack of trauma, a CT angiogram and coagulation profile were ordered. CTA of the head and neck was completed, which also showed an incidental left posterior cerebral artery aneurysm. The prothrombin time test/international normalized ratio, partial thromboplastin time test, and complete blood count laboratory results were within normal values. Given the CT findings, the patient was admitted to the neurological intensive care unit (NICU) with strict blood pressure monitoring and hourly neurological evaluations. Repeat CT imaging six hours after the initial scan showed stable results. Since there was no history of trauma, a diagnostic cerebral angiogram was performed to evaluate for a vascular etiology of the hemorrhage. Digital subtraction angiography (DSA) showed no additional findings besides the previously noted left posterior cerebral artery aneurysm. Given her stable clinical status, the patient was monitored in the ICU for three days with a daily CT scan of the head imaging, all displaying signs of stability. As such, the patient was transferred out of the ICU. On hospital day 4, the patient underwent a CT scan of the head prior to discharge, which illustrated a stable acute subdural hematoma. The patient was then discharged with plans for a repeat CT scan of the head in one week to follow up on the subdural hematoma and likely delayed surgical intervention.

**Figure 2:**
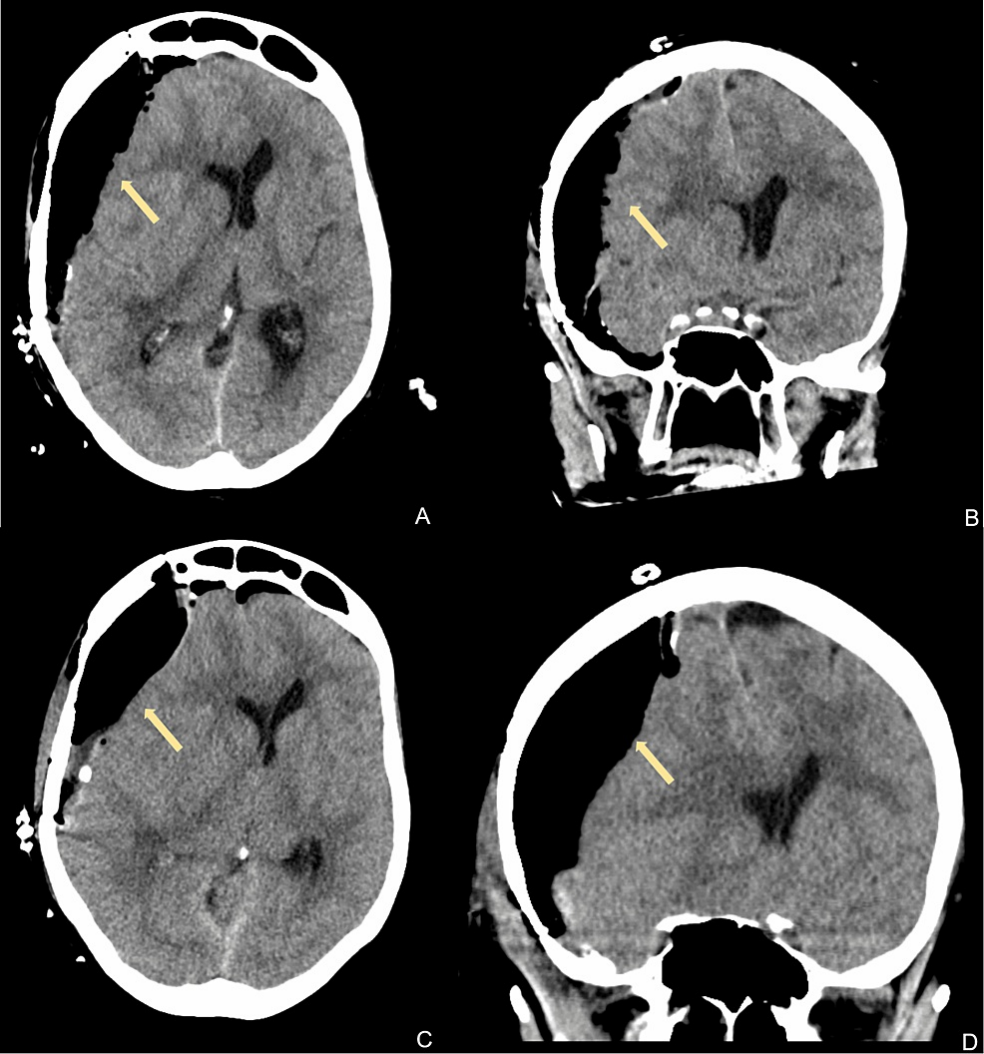
Computed tomography imaging of the head after subdural hematoma evacuation (arrows) The top row shows axial (A) and coronal (B) views on postoperative day 1. The bottom row shows axial (C) and coronal (D) views on postoperative day 2.

Two days after discharge, the patient then re-presented to the emergency room after a fall with increased left hemi-body weakness and decreased alertness. On examination, the patient had a GCS score of 10 and followed commands. CT scan of the head showed an interval increase in the right-sided subdural hematoma with a 1.2 cm shift (Figure 2). Due to the imaging findings, the patient was emergently rushed into the operating room for a right-sided subdural hematoma evacuation. During the operation, a right-sided craniotomy was performed with inadvertent extension into the right frontal sinus. The dura was then opened in a cruciate fashion where the subdural hematoma was evacuated circumferentially until the brain relaxed. The bone was plated back, and the muscles/skin was reapproximated. Postoperatively, a subdural drain was left in place and was attached to a Buretrol bag to facilitate passive drainage of any residual subdural contents. Postoperative CT scan of the head showed resolution of the hemorrhage with midline shift and pneumocephalus (Figure 2).

Postoperatively, the patient remained intubated. On examination, she was awake and alert and capable of following commands with her right upper extremity and bilateral lower extremities with spontaneous left upper extremity movement. Given the midline shift and pneumocephaly, a repeat CT scan of the head was ordered on postoperative day (POD) 1, which showed stable pneumocephaly and midline shift. The patient was extubated the same day given her stable imaging and examination. However, on the evening of POD 2, the patient began to decline in mentation and was re-intubated. A CT scan of the head was ordered, which showed an increase in the pneumocephalus and a worsening midline shift of 1.6 cm. At that point, the thought was that the subdural drain may have been leaking air into the subdural space, and thus, it was removed. Over the subsequent days, the patient’s neurological examination slowly continued to worsen with fluctuating alertness.

**Figure 3:**
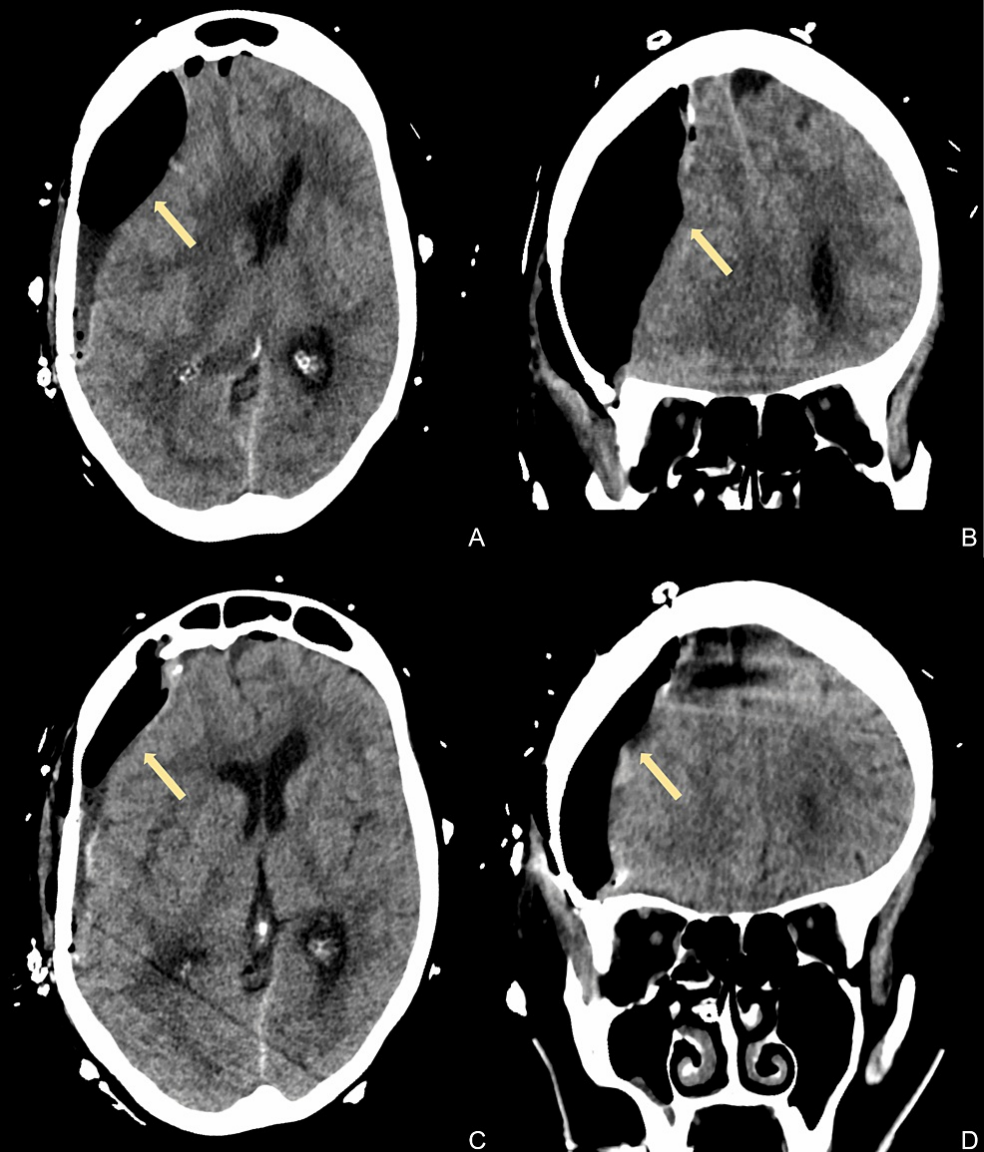
Computed tomography imaging of the head after subdural hematoma evacuation (arrows) The top row shows axial (A) and coronal (B) views on postoperative day 4. The bottom row shows axial (C) and coronal (D) views on postoperative day 4 immediately after subdural evacuating port system placement.

**Figure 4:**
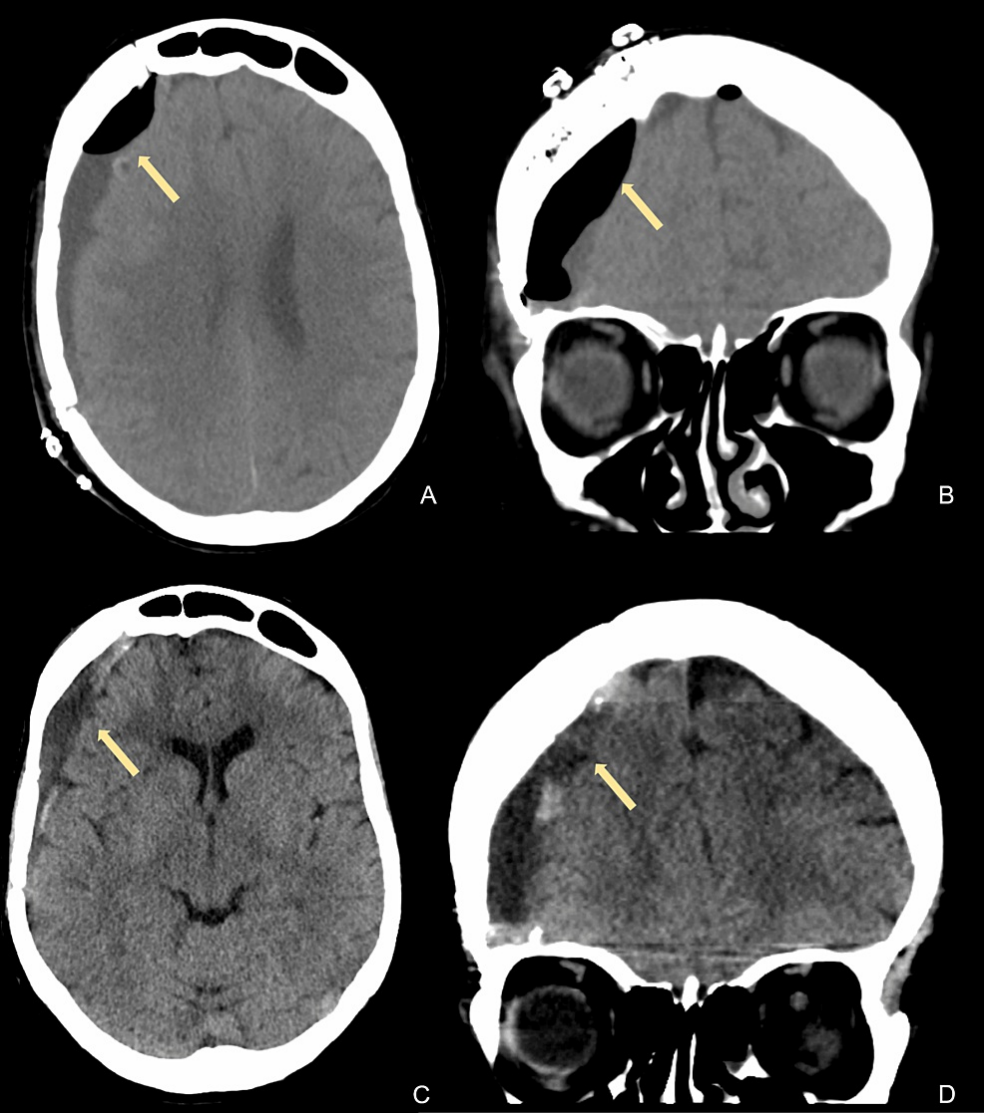
Computed tomography imaging of the head after subdural hematoma evacuation (arrows) The top row shows axial (A) and coronal (B) views on postoperative day 8 immediately prior to subdural evacuating port system removal. The bottom row shows axial (C) and coronal (D) views three months postoperatively.

Due to the fluctuating neurological examination, persistent pneumocephalus, and midline shift, a right-sided subdural evacuating port system (SEPS) drain was placed on POD 4. During the procedure, evidence of the release of air under high pressure from the subdural space was encountered. CT scan of the head post-SEPS placement showed a slight interval increase in subdural fluid collection and overall decreased midline shift and pneumocephalus (Figure 3). The patient’s examination post-SEPS improved, consistently following commands symmetrically. Given the improved examination, the patient was extubated on POD 5. The patient’s examination at that time was GCS 15 with motor strength of 4+/5 left upper extremity weakness and an otherwise neurologically non-focal examination. Repeat CT imaging showed improvement of the pneumocephalus with an improvement of midline shift (Figure 4). The patient was subsequently transferred to the inpatient hospital unit on POD 7, and the SEPS drain was removed. While there, the patient was placed on sinus precautions due to the concern that the frontal sinus defect had contributed to the tension pneumocephalus. On POD 9, after working with physical and occupational therapy, she was deemed stable for discharge to inpatient rehabilitation. Repeat imaging/visits three months and six months postoperatively showed improvement for both the pneumocephalus and clinical presentation.

## Discussion

Pneumocephalus describes the presence of air within the intracranial space. It can be classified as simple or tension and additionally as acute or delayed [1]. The development of pneumocephalus manifests through a variety of central nervous system disturbances such as penetrating skull injuries with involvement of the dura, irritation of the meningeal layers by gas-forming organisms, and epidural and subarachnoid blocks [1]. The incidence of pneumocephalus varies based on the causal factor, ranging from 3.9% to 9.7% [7-9]. Some common features of pneumocephalus that should increase clinical suspicion include postsurgical seizures, meningitis, tinnitus, and flapping scalp sign [10,11].

A plethora of imaging modalities are available for the diagnosis and management of pneumocephalus. Skull X-rays can be utilized; however, this modality has low sensitivity and is suboptimal due to its tendency of missing small pockets of air during imaging [1]. Magnetic resonance imaging (MRI) can also be utilized; however, this modality can have a tendency to mistakenly classify air for flow voids or blood products, yielding a dark appearance in the associated imaging sequence [1]. The standard bearing imaging modality for the diagnosis of pneumocephalus is a plain head CT [1]. It has been shown to detect very minuscule values of air, reportedly even 0.55 ml, while a skull radiograph requires a baseline of at least 2 ml of air [1].

Tension pneumocephalus is a potentially life-threatening complication that presents with nonspecific symptoms such as headache, coma, and hemiparalysis, alongside deteriorating GCS, and it requires emergent decompression [12,13]. A few theories that highlight the development of tension pneumocephalus include the “ball valve” theory of Dandy and the inverted bottle cap effect theory. The ball valve theory posits that pneumocephalus arises via a unidirectional movement of air from the external environment into the cranial cavity [1]. The inverted bottle cap theory postulates that trauma within the cranium leads to decreased intracranial pressure, resulting in a vacuum that causes an inflow of air [1]. Volume of as little as 6.5 ml of air has been demonstrated to be discernible enough to cause tension pneumocephalus [12]. The treatment of tension pneumocephalus arises from a timely recognition of its classic imaging phenomena, known as the “Mount Fuji” sign on a CT of the brain [14]. However, it is noteworthy that the “Mount Fuji” sign appears for bilateral tension pneumocephalus, which was not the case in this presentation. Common modes of treatment for tension pneumocephalus mentioned in the literature include controlled decompression using a closed water-seal drainage system, burr hole aspiration of air, needle aspiration, craniotomy, and ventriculostomy placement [6,15]. Our case represents one of the first methods of tension pneumocephalus decompression being managed using a SEPS drain.

A subdural evacuating port system is comprised of a stainless-steel evacuating port, a silicone tubing, and a bulb section driver [16]. SEPS drain placement is typically performed at the bedside of the NICU or in the emergency room. After sedation and local anesthesia, a skin incision is made, and a subsequent burr hole is placed using a mechanical hand drill. The dura is then opened. Following this procedure, a metal port is screwed into the hole within the cranium to allow the tip to be inserted within the medullary cavity of the skull. The external component of the evacuating port is then engaged with the silicone tubing and bulb suction. Negative pressure is applied using the supplied bulb for a variable time period until the drainage of subdural fluid is minimal [16].

Conventional operative treatments for tension pneumocephalus consist of open craniotomy in the operating room for the evacuation of intracranial air [6]. However, over the past decade, drainage systems such as SEPS drains have been becoming increasingly popular due to their potential in decreasing the utilization of systemic anesthesia and lower infection risk [17]. Additionally, SEPS drains have also been associated with a low postoperative infection rate and comorbidity rate, reduced seizure probability, and lowered hospitalization costs and length of stays when used for the evacuation of subdural hematomas. These characteristics are especially beneficial for elderly patients, who are known to be suboptimal surgical candidates [17,18]. Given the favorable characteristic profile of SEPS compared to surgical decompressive modalities, further inquiry should be pursued to analyze the feasibility of establishing SEPS as a favorable treatment alternative to open surgical decompression for iatrogenic tension pneumocephalus.

## Conclusions

The diagnosis and treatment of TP require the collaboration of the entire care team. Patients who have craniotomies after recent trauma to the head should be closely monitored by nurses. Significant alterations in mental status should prompt the attention of a physician, and imaging should be ordered. As TP is better understood and documented, takeback craniotomies may no longer be the gold standard for decompression. In this case, we showed the efficacy of a less invasive bedside procedure, the SEPS.
